# Parent–Adolescent Conflict across Adolescence: Trajectories of Informant Discrepancies and Associations with Personality Types

**DOI:** 10.1007/s10964-019-01054-7

**Published:** 2019-06-26

**Authors:** Stefanos Mastrotheodoros, Jolien Van der Graaff, Maja Deković, Wim H. J. Meeus, Susan Branje

**Affiliations:** 1 Department of Youth and Family, Faculty of Social and Behavioral Sciences, Utrecht University, Utrecht, The Netherlands; 2 Department of Clinical Child and Family Studies, Faculty of Social and Behavioral Sciences, Utrecht University, Utrecht, The Netherlands

**Keywords:** Parent–adolescent conflict, Parent–adolescent discrepancies, Personality types, RUO typology, Parenting

## Abstract

Parent–adolescent conflict can be intense, yet parents and adolescents do not always agree on the intensity of conflict. Conflict intensity tends to change during adolescence and is thought to be an indicator of how the parent–adolescent relationship transforms. However, parents and adolescents might differently perceive change in conflict intensity, resulting in changing discrepancies in conflict intensity throughout adolescence. Also, personality characteristics of parents and adolescents might affect the extent to which there are discrepancies in perceptions of conflict intensity. This multi-informant longitudinal study investigated a) the trajectories of parent–adolescent conflict intensity, b) the trajectories of informant discrepancies, and c) the prediction of these trajectories by parental and adolescent personality. Dutch adolescents (*N* = 497, 43.1% female, *M*_age_ = 13.03 at T1), their mothers, and their fathers reported on parent–adolescent conflict intensity and personality for six years. Latent Growth Curve Modeling and Latent Congruence Modeling revealed curvilinear changes in conflict intensity, as well as in discrepancies thereof. Two cycles of discrepancies emerged. First, in early-to-middle-adolescence discrepancies in perceptions of parents and adolescents increased, reflecting that adolescents’ perceived conflict intensity increased. Second, in middle-to-late-adolescence, father–adolescent discrepancies increased further, reflecting that fathers’ perceptions of conflict decreased. Resilient adolescents, mothers, and fathers reported lower levels of conflict intensity than Undercontrollers and Overcontrollers, but personality was not associated with the rate of change in conflict intensity. Finally, undercontrolling fathers and overcontrolling adolescents showed higher father–adolescent discrepancies. This study showed that parents and adolescents differentially perceive conflict intensity and that in the adolescent–father relationship, the extent of the differences depends on adolescent and father personality.

## Introduction

Conflicts among parents and adolescents may be among the most aggravating family experiences of adolescence, for parents and adolescents alike. However, parent–adolescent conflict can also forge change towards greater egalitarianism in family relationships (Branje 2018), and it is, therefore, important to better understand how parent–adolescent conflict develops across adolescence. Even though many studies show a decrease in conflict *frequency* throughout adolescence (Shanahan et al. 2007), conflict *intensity* tends to increase from early to middle adolescence and to decrease thereafter (De Goede et al. 2009). As most of what is known thus far is based on adolescents’ views of conflict intensity, a more coherent picture of how conflict intensity develops across adolescence can be achieved by taking the views of mothers and fathers into account. Also, investigating parent–adolescent discrepancies in conflict intensity through a developmental lens may provide additional insight into how parent–adolescent relationships change during adolescence (Korelitz and Garber 2016).

Research has shown that there is heterogeneity in the trajectories of conflict intensity and parent–adolescent discrepancies. Some parent–adolescent dyads have high conflict intensity and increase in conflict intensity over time, whereas other parent–adolescent dyads have lower and stable levels of conflict intensity across adolescence (Hadiwijaya et al. 2017). Because people’s personality affects the quality of their interpersonal relationships, as well as their perception and interpretation of these relationships, it is expected that personality is related to parent–adolescent conflict intensity (Mund et al. 2018), and parent–adolescent discrepancies in perceptions of conflict intensity. The aim of this multi-informant longitudinal study was threefold. The first aim was to investigate the trajectories of conflict intensity across adolescence from the perspective of mothers, fathers, and adolescents. The second aim was to investigate the trajectories of parent–adolescent discrepancies in perceptions of conflict intensity. The third aim was to test whether maternal, paternal, and adolescent personality predicted these developmental trajectories.

### Parent–Adolescent Conflict

During adolescence, young people are thought to develop more autonomy than during childhood (e.g., Branje et al. 2012). For adolescents to become competent in adult roles, parents need to gradually release part of their authority and allow the parent–adolescent relationship to transform towards a more egalitarian one (Branje et al. 2012). Adolescents generally expect independent decision making earlier than parents are willing to grant it (Deković et al. 1997), and this may create a fertile ground for parent–adolescent conflicts (Collins and Laursen 1992). Furthermore, adolescence usually coincides with parents’ midlife, an often challenging life stage, characterized by a need to re-evaluate their life course, adjust to new work conditions, and redefine life satisfaction (van Aken et al. 2006). Thus, a “coincidental” crisis emerges (Steinberg and Silk 2002), with increased conflict potential. In addition, the emotional repercussions of puberty (Cservenka et al. 2015), coupled with the still-developing emotional self-regulation during adolescence (Bowers et al. 2011) may increase the intensity of parent–adolescent conflicts (Laursen et al. 1998). Given that parent–adolescent conflict has significant consequences for adolescent adaptation (Branje et al. 2009), it is vital to study its developmental course throughout adolescence.

Empirical findings on the trajectory of parent–adolescent conflict intensity are inconsistent. Conflict intensity has been found to decrease from age 11 to age 12 and to remain stable until age 14 (Galambos and Almeida 1992). However, other longitudinal studies found that, on average, adolescent-reported conflict intensity increased between age 11 and age 15 (De Goede et al. 2009; McGue et al. 2005), and decreased between ages 16 to 19 (De Goede et al. 2009). Also, both studies found gender differences, with girls increasing in parent–adolescent conflict intensity more than boys (De Goede et al. 2009; McGue et al. 2005). A study on the development of adolescent-reported conflict intensity between ages 14 and 17 also revealed heterogeneity in development, with stable low negativity for most adolescents, and increasing negativity for a minority of adolescents (Seiffge-Krenke et al. 2010). To conclude, some empirical studies on the trajectories of conflict intensity showed an increase, and some showed stability. This inconsistency might be due to the different age periods examined in the various studies.

Furthermore, extant studies usually relied on a single informant (either the adolescent or a parent), leaving a knowledge gap on how parent–adolescent conflict intensity develops according to adolescents, mothers, and fathers. Parents and adolescents tend to perceive interpersonal conflict (Van Lissa et al. 2015), and aspects of their relationship (Mastrotheodoros et al. 2018), differently. Research on a related concept, conflict engagement, documented a temporary increase from early to middle adolescence only according to adolescents’ views, but not according to mothers’ and fathers’ views (Van Doorn et al. 2011). These reporter differences might result from the different position in the hierarchical relationship, in which adolescents want to acquire more autonomy, and parents are motivated to preserve the status quo.

### Parent–Adolescent Discrepancies

A gold standard in the measurement of psychosocial constructs in developmental research is the use of multiple informants. Using multiple informants not only provides a more well-balanced representation of conflict intensity trajectories but also allows investigating possible discrepancies in perceptions of conflict (De Los Reyes and Kazdin 2005). Such discrepancies are not measurement error (De Los Reyes et al. 2010), and instead may reflect key dynamics in the parent–adolescent relationship that are meaningfully associated with adolescent and parent adaptation (De Los Reyes et al. 2019). For example, differences among parental and adolescent views of conflict were positively associated with adolescent depressive symptoms and risky behaviors (Skinner and McHale 2016). Furthermore, adolescents in families with high parent–adolescent discrepancies in perceptions of family functioning were at higher risk for sexually dangerous behaviors and alcohol use (Córdova et al. 2016). In contrast, maternal psychological symptoms were higher when both mothers and adolescents agreed on low family satisfaction, compared to when only the mother reported low family satisfaction, but the adolescent reported high family satisfaction (Ohannessian et al. 2016). These findings show that taking more than one perspective into account might be more insightful compared to using only one informant.

Parent-child discrepancies might be particularly relevant to investigate during adolescence. The stage-environment fit hypothesis (Eccles et al. 1993) posits that the opportunities offered by the social context adolescents live in (e.g., the family) might not fit adolescents’ developmental needs. Adolescents show increasing decision-making abilities and need for autonomy, and parents may not always optimally recognize and respond to these changing needs. Such a “needs-opportunities” mismatch might be reflected in parent–adolescent discrepancies in the perception of the quality of their relationship. Besides, as adolescents grow older, they may refrain from disclosing information to their parents, as a means to establish autonomy (Keijsers et al. 2009). This decreased disclosure results in less communication, which has been associated with increased parent–adolescent discrepancies (Ehrlich et al. 2016). However, towards late adolescence, the parent–adolescent relationship becomes more egalitarian (Branje 2018), and therefore, increasing agreement (i.e., less discrepancy) in perceptions may be expected. This curvilinear trend in discrepancies across adolescence is in line with the Operations Triad Model (De Los Reyes and Ohannessian 2016), which suggests that a curvilinear pattern, where discrepancies initially increase from early to middle adolescence and decrease thereafter, might indicate normative development. However, whether such a pattern exists, and whether this is the normative pattern of discrepancy development during adolescence can only be clarified by adopting a developmental perspective on parent–adolescent discrepancies (De Los Reyes et al. 2019). Hence, investigating parent–adolescent discrepancies developmentally has been recently ranked as the main priority in parent–adolescent discrepancy research (De Los Reyes et al. 2019).

Despite the emerging literature on parent–adolescent discrepancies (De Los Reyes and Ohannessian 2016), few studies have investigated how discrepancies develop during adolescence. Extant studies showed that discrepancies could develop in different directions. A meta-analysis on cross-sectional studies on the degree and direction of parent-child discrepancies in parenting constructs revealed moderation by age, such that in samples with older children there was less parent-child discrepancy than in samples with younger children (Korelitz and Garber 2016). A recent one-year longitudinal study found that mother–adolescent discrepancies in perceptions of open communication increased between ages 16 and 17, but discrepancies in perceptions of communication problems did not (De Los Reyes et al. 2016). Additionally, another study found a curvilinear pattern of parent–adolescent differences in perceptions of familism from age 12 to age 22, where an initial increase was followed by a decrease (Padilla et al. 2016). Finally, a study that investigated the heterogeneity in the trajectories of parent–adolescent discrepancies in family functioning found that for most families the discrepancies were low and stable across adolescence, yet a minority of families was characterized by either high stable or high increasing discrepancies (Córdova et al. 2016). However, the developmental trajectories of mother–adolescent, and father–adolescent discrepancies in conflict intensity across adolescence remain largely unknown.

### Personality Types and Parent–Adolescent Conflict

Interpersonal relationships vary in quantity and quality, and this is partly because of the personalities of the dyad members (e.g., Mund and Neyer 2014). Because personality and interpersonal relationships are linked (e.g., Mund and Neyer 2014), heterogeneity in parent–adolescent conflict intensity (De Goede et al. 2009), as well as in its development (Seiffge-Krenke et al. 2010), may stem from parental or adolescent personality. In the current study, a typological approach to personality was applied (Asendorpf and van Aken 1999), which recognizes that people employ a constellation of characteristics instead of single, segregated characteristics in isolation (Yu et al. 2014). One of the most commonly applied person-centered approaches to personality is Block and Block’s RUO (Resilients, Undercontrollers, Overcontrollers) typology (Block and Block 1980). Based on this typology three personality types have been proposed: Resilient, characterized by relatively high scores on all Big Five factors; Undercontrollers, mainly characterized by low Agreeableness and Conscientiousness; and Overcontrollers, mainly characterized by low Emotional Stability, low Extraversion, and average or high scores on the other three dimensions (Klimstra et al. 2009a).

Adolescents and adults with an undercontrolling or overcontrolling personality type may employ more conflictual behaviors in their relationships. Undercontrollers and Overcontrollers are characterized by personality characteristics that are typically associated with more conflictual relationships. That is, they tend to have characteristics that relate to higher anger and aggression, like lower Agreeableness (De Fruyt et al. 2017; Jensen-Campbell and Graziano 2001), and lower Conscientiousness (Jensen-Campbell and Malcolm 2007) for Undercontrollers, and lower Emotional Stability (Jones et al. 2011) for Overcontrollers. Undercontrolling children (Denissen et al. 2007) and adults (Bohane et al. 2017) indeed show higher levels of aggression and engagement in conflicts compared to Resilients. Furthermore, personality, by definition, encompasses differences in how people perceive their environment (Mund and Neyer 2014). Therefore, undercontrolling or overcontrolling adolescents and adults may also view their interpersonal relationships differently compared to how their partners perceive them. This might give rise to higher discrepancies. For example, Undercontrollers, who have low Agreeableness and Conscientiousness, and Overcontrollers, who have low Emotional Stability, may be more difficult to relate and openly communicate with, which may lead to higher divergence in relational perceptions. Indeed, mothers with higher trait negative affectivity and lower trait positive affectivity show higher discrepancies with their sons, compared to mothers with a more adaptive profile (Shishido and Latzman 2017). Resilients might not only have more adaptive and less conflictual relationships, but might also more easily and straightforward communicate about their relationship perceptions and have a higher mutual understanding. This might result in lower discrepancies in perceptions. Therefore, adolescents and parents with a Resilient personality type are expected to have lower conflict intensity and lower discrepancies than Overcontrollers and Undercontrollers.

## The Present Study

Taken together, extant research has shown that adolescents’ views of parent–adolescent conflict intensity display a curvilinear trend across adolescence. However, less is known about the development of conflict intensity across adolescence according to mothers and fathers. More importantly, trajectories of parent–adolescent discrepancies remain understudied, and so do possible determinants of such trajectories. The current multi-informant and longitudinal study aimed to answer the following questions: How does parent–adolescent conflict intensity develop across adolescence, according to the views of mothers, fathers, and adolescents? (RQ1) Given previous studies that found a curvilinear trend of conflict intensity (e.g., De Goede et al. 2009), it was expected that conflict intensity would increase from early to middle adolescence, and decrease thereafter (Hypothesis 1). How do parent–adolescent discrepancies in conflict intensity develop across adolescence? (RQ2) Based on theoretical perspectives (De Los Reyes and Ohannessian 2016; Eccles et al. 1993), it was expected that an initial increase would be followed by a decrease in discrepancies in perceptions of conflict intensity (Hypothesis 2). Do personality types predict the trajectories of conflict intensity and the trajectories of discrepancies in perceptions of conflict intensity across adolescence? (RQ3) Given the role personality types play in interpersonal interaction (Denissen et al. 2007) and informant discrepancies (e.g., Shishido and Latzman 2017), it was expected that a Resilient personality type would be associated with lower conflict intensity, and smaller parent–adolescent discrepancies (Hypothesis 3).

## Method

### Participants

The sample consisted of 497 adolescents (43.1% girls, *M*_age_ = 13.03, *SD* = 0.46, at T1; *M*_age_ = 18.03, *SD* = 0.46, at T6), their mothers (N = 497, *M*_age_ = 40.41, *SD* = 4.45, at T1), and their fathers (*N* = 456, *M*_age_ = 46.74, *SD* = 5.11, at T1) who took part in six annual assessments of an ongoing longitudinal study (Research on Adolescent Development And Relationships, see https://www.uu.nl/en/research/radar) in The Netherlands, from 2006 to 2011. Adolescents were recruited from randomly selected elementary schools from the province of Utrecht as well as from three other big cities in The Netherlands. From a list of 850 regular schools in the western and central regions of the Netherlands, 429 were randomly selected and approached. Of those, 296 (69%) were willing to participate, and 230 of those were approached. Schools were used for initial screening (teacher reports for all 12-year-old students), as well as a means to approach families. Of the total of students screened (*n* = 4615), 1544 were randomly selected. Because the aim of the study was to include two-parent families with at least one more child older than 10 years old, 1081 families were approached. Of those, 470 refused to take part and 114 did not sign informed consent, resulting in the final sample of 497 families.

Data were collected via annual home visits during which participants filled-in self-report questionnaires, and procedures were the same for all six waves. During the first measurement wave, adolescents were in 7th Grade. Most adolescents were native Dutch (94.8%) and lived with both parents (85.2%). Regarding parental occupation, for 87.7% of adolescents at least one of the parents’ jobs was classified as medium level (e.g., police officer, physician’s assistant) or high level (e.g., doctor, scientist, high school teacher), whereas 12.3% of adolescents came from families in which parents were either unemployed, or held an elementary job (e.g., construction worker, janitor, truck driver; Statistics Netherlands 1993). Furthermore, most parents had completed either secondary (55.9% of mothers; 48.1% of fathers) or higher education (40.2% of mothers; 49.6% of fathers).

### Measures

#### Parent–adolescent conflict intensity

To measure conflict intensity, 6 items from the Negative Interactions scale, from the Network of Relationships Inventory—short form (NRI) were used (De Goede et al. 2009; Furman and Buhrmester 1985). Participants answered on a 5-point Likert scale, ranging from 1 (*Little or Not at all*) to 5 (*More is not possible*) how much anger, irritation, and negative behaviors they experienced in their relationship. The scale was completed by (a) adolescents regarding the relationship with their mother (Adolescent-Mother report, AM); (b) adolescents regarding the relationship with their fathers (Adolescent-Father report, AF); (c) mothers regarding the relationship with the adolescents (mother–adolescent report, MA); and (d) fathers regarding the relationship with the adolescents (father–adolescent report, FA). Cronbach’s α’s ranged across waves between α’s = 0.90–0.95 (adolescent-mother report); α’s = 0.90–0.94 (adolescent-father report); α’s = 0.90–0.92 (mother–adolescent report); α’s = 0.90–0.92 (father–adolescent report). Example items are: “How much do you and your mother/father/child get upset with or mad at each other?” and “How much do you and your mother/father/child get on each other’s nerves?”

#### Personality

To measure maternal, paternal, and adolescent personality, the shortened Dutch version of Goldberg’s Big Five Questionnaire was used (Goldberg 1992; Vermulst and Gerris 2005). This questionnaire applies a 7-point Likert scale with a response format ranging from 1 (*Completely untrue*) to 7 (*Completely true*), to assess five personality dimensions: Extraversion, Agreeableness, Conscientiousness, Emotional Stability, and Openness to Experience. It consists of 30 adjectives, six per personality trait, such as “imaginative” (Openness to Experience), “organized” (Conscientiousness), “talkative” (Extraversion), “sympathetic” (Agreeableness), and “worried” (Emotional Stability, reverse coded). Mothers, fathers, and adolescents addressed these adjectives with reference to themselves. Previous studies have shown that this instrument has adequate reliability and validity when administered among adolescents (Klimstra et al. 2009b). In the current study the Cronbach’s *α*’s across waves and across the Big Five dimensions ranged between α’s = 0.72–0.89 (adolescent reports), α’s = 0.84–0.91 (mother reports), and α’s = 0.79–0.91 (father reports).

### Attrition and Missing Values

The majority of adolescents (85.7%), mothers (84.5%), and fathers (75.5%) were still involved in the study at Wave 6, and the average participation rate across the six waves was 90.4, 90.2, and 81.7%, for adolescents, mothers, and fathers, respectively. Little’s MCAR test (Little 1988) was significant [*χ*^2^ (8308) = 9216, *p* = 0.000], but the normed *χ*^2^/df (9216/8308 = 1.11) indicated that the assumption of missingness being completely at random was not seriously violated. Therefore, data from all 497 families could be included in the analyses using Full Information Maximum Likelihood.

### Procedure

The study was approved by the medical ethics committee of Utrecht University (METC). Before the start of the study, parents were required to provide informed consent, and adolescents to provide assent. Adolescents and parents filled out questionnaires during annual home visits. Trained research assistants provided verbal instructions in addition to written instructions that accompanied the questionnaires. Confidentiality was guaranteed, and the data were processed anonymously. Each wave families received 100 euros for their participation.

### Analytic Plan

The first analytic step consisted of testing measurement invariance of the Negative Interactions scale across the four reports, within each wave (Van de Schoot et al. 2012) using MPlus 8.2 (Muthén and Muthén 1996–2018). To account for dependency in the observations, Type=COMPLEX and a unique family code as clustering variable were used. After specifying a univariate model in which the six items loaded on one latent factor, the function automatically estimated a configural, a metric, and a scalar invariance model, corresponding to equality of the factor structure, the item loadings, and the item intercepts and loadings, respectively. A maximum likelihood estimator with robust standard errors was chosen, given the right-skew of the scale. Also, for each reporter, longitudinal measurement invariance of the Negative Interactions scale across the six waves was tested, using the meas Eq.syntax function of the semTools package in R (Jorgensen et al. 2018).

To answer the first research question on the trajectories of parent–adolescent conflict intensity across adolescence, four univariate Latent Growth Curve Models (LGCM, Wang and Wang 2012) were applied in lavaan (Rosseel 2012), separately for the AM, AF, MA, and FA reports. For each model, it was first examined whether linear or quadratic slope fit the data best, based on model fit indices (CFI, TLI, RMSEA, SRMR, and BIC). To ease comparisons and interpretation, in case the quadratic slope fit the model better compared to the linear, piecewise LGCM was applied. For that purpose, a series of LGCM was run, where the “knot,” that is, the point the slope would be split into two linear pieces, was tested in different time points. These models were compared in terms of model fit (CFI, TLI, RMSEA, and SRMR), and the model that fit the data best was selected as the piecewise model of choice.

To answer the second research question, regarding the development of parent–adolescent discrepancies in conflict intensity, the following steps were applied. First, Latent Congruence Modeling (Cheung 2009; Ksinan and Vazsonyi 2016) was used to estimate two latent factors, based on two reports (e.g., AM and MA reports). The latent mean level factor captures the mean of the two reports, and each of the two reports has a factor loading of 1 on this factor. The latent congruence factor captures the latent difference of the two reports by constraining the first reporter’s (here, the adolescent’s) loadings to 0.5 and the other reporter’s (here, the parent’s) to −0.5. The latent congruence factor was estimated separately for each dyad (mother–adolescent; father–adolescent) and each year of measurement (T1–T6). For each of these models, the latent congruence factor scores were saved. Second, LGCMs were applied on the saved scores for mother–adolescent and father–adolescent dyads separately. For the LGCMs, the same steps as those regarding answering the first research question were followed.

To answer the third research question on the role of personality types, the growth patterns of the Big Five were first investigated separately for adolescents, and parents, to determine the shape of the curve, as well as the existence of significant variance around the mean estimates. Next, Latent Class Growth Analysis (LCGA, Jung and Wickrama 2008; Nagin 2005) was applied in M*plus* 8.2 (Muthén and Muthén 1996–2018) on the resulting trajectories of the Big Five traits, separately for adolescents and parents, as a means to investigate different personality type trajectories of adolescents and parents. Gender was controlled for, to account for the gender differences in personality (Klimstra et al. 2009a). The number of classes was decided based on both theoretical and empirical grounds. Theoretically, a three-class solution was expected, resembling Block and Block’s (1980) RUO typology. On the empirical level, a lower sample-adjusted Bayesian Information Criterion (BIC), a higher entropy (classification accuracy), and a significant Vuong-Lo-Mendel-Rubin Likelihood Ratio Test (VLMR-LRT) were used as criteria for the number of classes. Third, the resulting latent personality classes were used as predictors of the intercepts and slopes of the four univariate LGCMs for conflict intensity (i.e., for the AM, AF, MA, and FA reports of conflict intensity), and the two LGCMs for the discrepancies in conflict intensity. Specifically, dummy variables were created for each personality type. The intercepts and the slopes of conflict intensity in the mother–adolescent relationship as reported by mothers and adolescents (in separate models) and discrepancies in mother–adolescent conflict intensity were regressed on adolescent and maternal personality types. Similarly, the intercepts and the slopes of conflict intensity in the father–adolescent relationship as reported by fathers and adolescents (in separate models) and discrepancies in father–adolescent conflict intensity were regressed on adolescent and paternal personality types.

## Results

### Descriptive Statistics

Table 1 provides the means, standard deviations, and bivariate correlations of all conflict intensity scores across waves.

**Table 1:** Means, standard deviations, and correlations for the longitudinal scores on the Negative Interactions scale

Variable	*M*	*SD*	SES	Gender	1	2	3	4	5	6	7	8	9	10	11	12	13	14	15	16	17	18	19	20	21	22	23
Adol.-Moth																											
1. neg1	1.66	0.59	0.14***	0.07																							
2. neg2	1.71	0.67	0.12**	0.07	0.56**																						
3. neg3	1.75	0.67	0.06	0.01	0.50**	0.61**																					
4. neg4	1.79	0.69	0.04	0.03	0.43**	0.58**	0.63**																				
5. neg5	1.80	0.72	0.00	0.02	0.38**	0.50**	0.59**	0.69**																			
6. neg6	1.74	0.65	−0.01	0.04	0.35**	0.45**	0.54**	0.62**	0.64**																		
Adol.-Fath.																											
7. neg1	1.51	0.56	0.03	−0.02	0.34**	0.29**	0.13**	0.13**	0.16**	0.16**																	
8. neg2	1.60	0.64	0.06	0.04	0.31**	0.40**	0.24**	0.24**	0.22**	0.17**	0.67**																
9. neg3	1.67	0.66	0.00	0.06	0.19**	0.27**	0.31**	0.18**	0.23**	0.19**	0.54**	0.61**															
10. neg4	1.70	0.67	−0.04	0.04	0.17**	0.22**	0.20**	0.27**	0.22**	0.24**	0.50**	0.54**	0.69**														
11. neg5	1.70	0.69	0.04	0.04	0.27**	0.32**	0.32**	0.39**	0.40**	0.30**	0.44**	0.51**	0.57**	0.63**													
12. neg6	1.69	0.68	−0.01	0.04	0.20**	0.20**	0.16**	0.27**	0.30**	0.32**	0.35**	0.43**	0.48**	0.63**	0.68**												
Mother																											
13. neg1	1.52	0.53	0.13***	0.15***	0.43**	0.44**	0.39**	0.38**	0.35**	0.31**	0.26**	0.24**	0.22**	0.18**	0.29**	0.15**											
14. neg2	1.55	0.54	0.11*	0.12**	0.39**	0.53**	0.43**	0.33**	0.32**	0.33**	0.23**	0.31**	0.24**	0.19**	0.31**	0.11*	0.69**										
15. neg3	1.52	0.50	0.06	0.11*	0.29**	0.43**	0.50**	0.36**	0.37**	0.33**	0.13**	0.22**	0.30**	0.18**	0.24**	0.07	0.58**	0.67**									
16. neg4	1.55	0.56	0.04	0.04	0.28**	0.40**	0.39**	0.47**	0.38**	0.36**	0.16**	0.19**	0.21**	0.21**	0.30**	0.16**	0.56**	0.59**	0.71**								
17. neg5	1.50	0.54	0.13**	0.08	0.27**	0.41**	0.39**	0.43**	0.49**	0.42**	0.20**	0.24**	0.25**	0.20**	0.35**	0.22**	0.56**	0.60**	0.66**	0.74**							
18. neg6	1.48	0.54	0.12*	0.07	0.27**	0.43**	0.43**	0.44**	0.46**	0.54**	0.15**	0.17**	0.23**	0.18**	0.31**	0.22**	0.51**	0.56**	0.64**	0.66**	0.71**						
Father																											
19. neg1	1.51	0.50	0.05	−0.04	00.16**	0.21**	0.10*	0.10*	0.11*	0.09	0.52**	0.41**	0.37**	0.32**	0.30**	0.21**	0.39**	0.35**	0.27**	0.29**	0.29**	0.20**					
20. neg2	1.52	0.53	0.08	0.04	0.10*	0.21**	0.11*	0.07	0.05	0.09	0.44**	0.49**	0.37**	0.33**	0.27**	0.19**	0.28**	0.41**	0.33**	0.30**	0.28**	0.24**	0.70**				
21. neg3	1.51	0.52	0.04	−0.02	0.11*	0.19**	0.19**	0.17**	0.08	0.12*	0.31**	0.37**	0.51**	0.43**	0.36**	0.23**	0.26**	0.33**	0.43**	0.37**	0.30**	0.26**	0.63**	0.67**			
22. neg4	1.53	0.51	−0.00	−0.02	0.12*	0.13*	0.11*	0.17**	0.08	0.11*	0.28**	0.28**	0.38**	0.45**	0.39**	0.28**	0.28**	0.28**	0.31**	0.41**	0.30**	0.24**	0.57**	0.61**	0.71**		
23. neg5	1.51	0.53	0.01	0.01	0.15**	0.18**	0.16**	0.23**	0.19**	0.21**	0.28**	0.28**	0.33**	0.40**	0.51**	0.37**	0.22**	0.32**	0.33**	0.40**	0.45**	0.41**	0.54**	0.51**	0.62**	0.72**	
24. neg6	1.47	0.49	0.07	−0.01	0.11*	0.14**	0.12*	0.23**	0.16**	0.22**	0.18**	0.24**	0.25**	0.31**	0.43**	0.39**	0.18**	0.22**	0.22**	0.26**	0.28**	0.40**	0.45**	0.46**	0.55**	0.58**	0.77**

*M* mean, *SD* standard deviation, *SES* socio-economic status, *Adol.-Moth.* adolescent report for mother, *Adol.-Fath*. adolescent report for father, *neg1–neg6* negative interaction score on Wave 1 through Wave 6

*p < 0.05; **p < 0.01; ***p < 0.001

### Measurement Invariance of the Negative Interactions scale

As seen in Table S1 (see Supplemental information), all models supported scalar invariance across reporters, for all waves, as well as across waves. Imposing restrictions for equality of factor loadings, and subsequently for item intercepts, did not lead to worse fit beyond the recommended thresholds (*Δ*CFI ≤ 0.010, *Δ*TLI ≤ 0.010, *Δ*RMSEA ≤ 0.015, Cheung and Rensvold 2002). Also, the scalar models were in all cases those with the lowest BIC, therefore achieving the best parsimony-to-fit balance among the three models (configural, metric, scalar).

### Development of Conflict Intensity across Adolescence

For all four Latent Growth Curve Models, a non-linear slope fit the data better compared to a linear slope (Table S2, in Supplemental information). Therefore, a series of piecewise LGCMs was applied, to detect the time point where the knot fit best (Wang and Wang 2012). The time where the knot fit the data best was Wave 3 (adolescent age 15 years) for adolescent-father reports, and Wave 4 (adolescent age 16 years) for adolescent-mother reports and mother- and father-reports of conflict intensity.

**Fig. 1:**
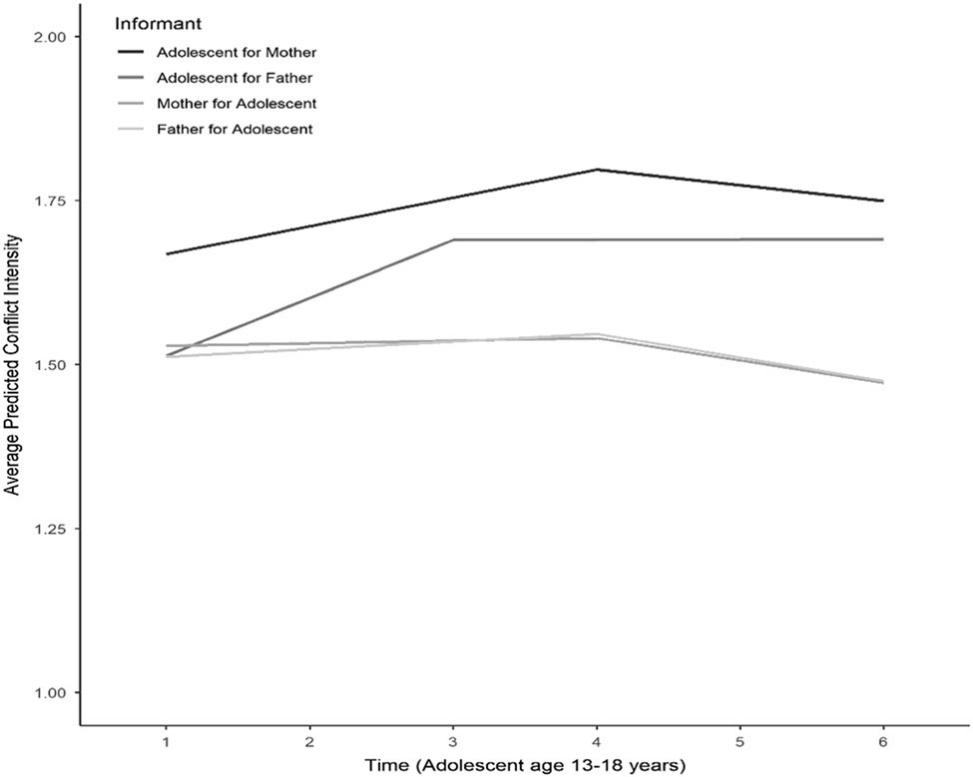
Developmental trajectories of adolescent-, and parent-reported conflict intensity across adolescence

Table 2 presents the intercepts and slopes for these four LGCMs (see also Fig. 1). According to adolescents, conflict intensity with their mothers and fathers increased from early to middle adolescence (ages 13 to 16 for adolescents’ relationship with their mothers, and ages 13 to 15 for adolescents’ relationship with their fathers), and then remained stable. According to mothers and fathers, conflict with their adolescents remained stable from early to middle adolescence (ages 13 to 16) and then decreased until age 18. For most intercepts and slopes, there was significant variance around the average estimates, which indicates that families differ in level and change in parent–adolescent conflict intensity.

**Table 2:** Growth parameter estimates (means and variances) of the latent growth curve models for adolescent-, mother-, and father-reported conflict intensity, and the latent growth curve models for mother–adolescent and father–adolescent discrepancies in conflict intensity

	Intercept		Slope 1		Slope 2	
	Mean	Variance	Mean	Variance	Mean	Variance
Conflict Intensity						
AM	1.67***	0.197***	0.045***	0.021***	−0.021	0.024
AF	1.51***	0.209***	0.090***	0.035***	0.003	0.027***
MA	1.52***	0.160***	0.007	0.009***	−0.036***	0.011
FA	1.51***	0.156**	0.013	0.009***	−0.039***	0.040***
Discrepancies						
Mother–Adolescent	00.140***	0.071***	0.034***	0.015***	0.017	0.021*
Father–adolescent	0.003	0.087***	0.072***	0.011**	0.019*	0.010***

*AM* adolescent report for mother, *AF* adolescent report for father, *MA* mother report for adolescent, *FA* father report for adolescent

*p < 0.05; **p < 0.01; ***p < 0.001

### Development of Parent–Adolescent Discrepancies in Conflict Intensity

Table S3 (in Supplemental information) presents the means and the variances of the Latent Congruency Models, for adolescent-mother, and adolescent-father dyads, across the six waves. The LGCMs on the saved congruence scores were used to investigate the development of parent–adolescent discrepancies in conflict intensity (Cheung 2009). Table S2 (in Supplemental information) presents the fit indices of models with linear, quadratic, and piecewise modeling of development. As non-linear growth showed a better fit compared to linear growth, a piecewise model to investigate latent change was fit, to ease interpretability.

**Fig. 2:**
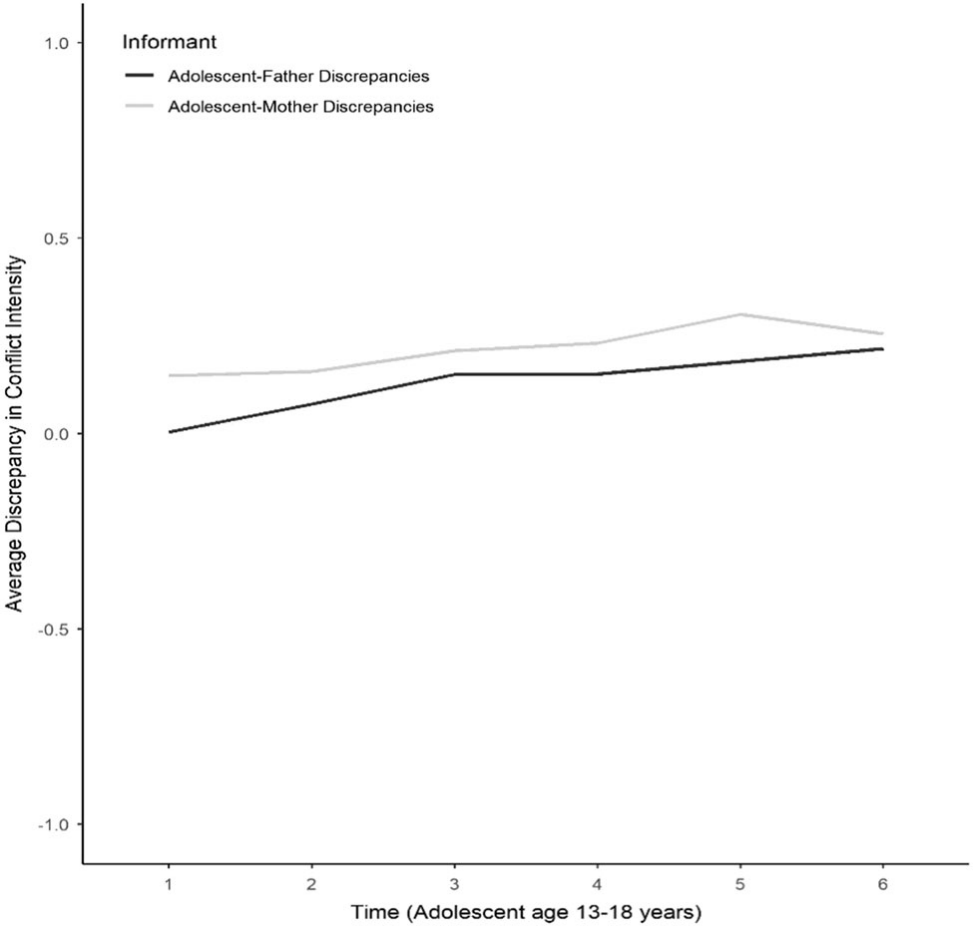
Developmental trajectories of adolescent-father, and adolescent-mother absolute discrepancies in conflict intensity across adolescence

Table 2 presents the means and variances of the latent intercepts and slopes for the development of mother–adolescent and father–adolescent discrepancies in conflict intensity (see also Fig. 2). In mother–adolescent dyads, the intercept was positive and significant, indicating that adolescents reported more conflict intensity than their mothers. The slope from Wave 1 (adolescent age 13 years) to Wave 4 (adolescent age 16 years) was positive and significant, indicating that, on average, mothers’ and adolescents’ perceptions of conflict intensity in their relationship further diverged in this age range. The slope from Wave 4 (adolescent age 16 years) to Wave 6 (adolescent age 18 years) was non-significant, indicating that, on average, mother–adolescent discrepancies remained stable in this age range. In father–adolescent dyads, the intercept was close to zero. That is, on average, fathers and adolescents held similar perceptions of conflict intensity in their relationship at Wave 1. However, both slope 1 (Wave 1 to Wave 3; adolescent age 13 years to 15 years) and slope 2 (Wave 3 to Wave 6; adolescent age 15 years to 18 years) were positive and significant, indicating that, over the course of adolescence, adolescents reported increasingly higher conflict intensity than fathers did. Two cycles that comprise this increasing divergence can be seen by inspecting the univariate LGCMs on adolescent-father and father–adolescent reports of conflict intensity. From early to middle adolescence (age 13 years to 15 years), the divergence emerged because adolescents perceived an increase in conflict intensity whereas their fathers reported stable levels of conflict, but from middle to late adolescence (age 15 years to 18 years), the divergence is due to the decline in perceptions of conflict intensity by fathers while adolescents reported stable levels of conflict intensity.

### Personality Types

To examine whether personality types could predict the development of conflict intensity and the heterogeneity thereof, personality types were first constructed. Table 3 presents the results of the Latent Class Growth Analyses (Nagin 2005). For both adolescents and parents, a solution with three classes was selected, upon theoretical, interpretative, and statistical grounds. For adolescents, a 3-class solution had lower BIC and higher entropy compared to a 2-class solution, and a significant VLMR-LRT. A 4-class solution had lower BIC and higher entropy, but a non-significant VLMR-LRT compared to the 3-class solution. Further, interpreting the four classes became difficult because two classes overlapped significantly (see also Figs S1–S2 in Supplemental information). Adolescents were roughly equally spread among the three classes: 183 adolescents (36.8%) in class 1, 156 (31.4%) in class 2, and 158 (31.8%) in class 3. Inspecting the mean intercepts and slopes of the Big Five of these classes (Fig. S1 and Table S4, in Supplemental information) led us to label them “Resilients,” “Overcontrollers,” and “Undercontrollers,” respectively. Resilients scored high on all Big Five factors. Overcontrollers scored the lowest on Emotional Stability and Extraversion, and Undercontrollers scored the lowest scores on Agreeableness, Conscientiousness, and Openness.

**Table 3:** Fit Indices for the latent class growth analyses models with different numbers of classes

	aBIC	Entropy	VLMR-LRT
Adolescent			
One class	38,322	–	–
Two classes	36,717	0.876	1656**
Three classes	35,550	0.892	1217*
Four classes	35,116	0.905	492
Five classes	34,765	0.886	404
Parents			
One class	71,631	–	–
Two classes	67,017	0.915	4655***
Three classes	65,076	0.918	1991
Four classes	63,406	0.916	1708
Five classes	62,304	0.916	1139

*aBIC* sample-adjusted Bayesian Information Criterion, VLMR-LRT Vuong-Lo-Mendel-Rubin Likelihood Ratio Test

*p < 0.05; **p < 0.01; ***p < 0.001

For parents, a solution with 3-classes showed lower BIC and higher entropy compared to a 2-class solution, and higher BIC but still higher entropy compared to a 4-class solution. Inspection of the 3- and the 4-class solutions showed overlap among classes in the 4-class solution (see also Figs S3 and S4 in Supplemental information). Inspecting the mean intercepts and slopes of the Big Five of these classes (Fig. S3 and Table S5, in Supplemental information), led us to label them “Resilients,” “Overcontrollers,” and “Undercontrollers,” respectively. Most parents (mothers: *n* = 245, 49.3%; fathers: *n* = 211, 46.2%) were in the Resilient class, with the rest being equally distributed as Overcontrollers (mothers: *n* = 135, 27.2%; fathers: *n* = 118, 25.8%), and Undercontrollers (mothers: *n* = 117, 23.5%; fathers: *n* = 128, 28%). Resilients scored significantly higher than the other two classes on all Big Five factors, and they also showed increasing levels on all factors, contrary to the other classes. Overcontrollers showed the lowest Emotional Stability, Extraversion, and Openness, whereas Undercontrollers showed the lowest Conscientiousness.

### Personality Types and the Development of Parent–Adolescent Conflict Intensity, and Discrepancies in Conflict Intensity

Three dummy variables were created, indicating the presence of each personality type. A series of analyses were run to compare Overcontrollers and Undercontrollers with Resilients. In these analyses, the intercepts and slopes of conflict intensity and discrepancies in conflict intensity were regressed on the dummy variables indicating Overcontrollers (0 = no, 1 = yes) and Undercontrollers (0 = no, 1 = yes). To compare Overcontrollers with Undercontrollers, a series of analyses were run using Resilient dummy and Undercontroller dummy as predictors.

Table 4 presents the regression coefficients of the intercepts of conflict intensity and discrepancies in conflict intensity on adolescent, maternal, and paternal personality. No significant results emerged regarding regressions of slopes, indicating that personality did not predict the rate of change in conflict intensity, and therefore, these results are omitted from the table.

**Table 4:** Regression coefficients (Unstandardized *Β*, and Standardized *β*) and confidence intervals for the prediction of the intercepts of conflict intensity and discrepancies in conflict intensity by adolescent and parental personality type

	Adolescent			Mother			Father		
	U versus R	O versus R	U versus O	U versus R	O versus R	U versus O	U versus R	O versus R	U versus O
Parent–adolescent conflict intensity									
AM intercept									
B	0.150	0.149	0.001	0.133	0.235	−0.102	–	–	–
C.I.	0.03–0.27	0.02–0.27	−0.14–0.14	0.01–0.26	0.11–0.36	−0.26–0.05	–	–	–
*β*	0.158**	0.157*	0.001	0.127*	0.234***	−0.097	–	–	–
MA intercept									
B	0.119	0.072	0.048	0.129	0.132	−0.003	–	–	–
C.I.	0.01–0.0.23	−0.04–0.18	−0.06–0.16	0.02–0.24	0.02–0.24	−0.13–0.13	–	–	–
*β*	0.139*	0.083	0.056	0.137*	0.148*	−0.004	–	–	–
AF intercept									
B	0.145	0.125	0.019	–	–	–	−0.019	0.043	−0.061
C.I.	0.03–0.26	0.01–0.25	−0.11–0.15	–	–	–	−0.14–0.10	−0.09–0.18	−0.21–0.08
*β*	0.147*	0.127*	0.020	–	–	–	−0.019	0.042	−0.062
FA intercept									
B	0.063	−0.032	0.095	–	–	–	0.123	0.147	−0.024
C.I.	−0.06–0.18	−0.13–0.07	−0.02–0.21	–	–	–	0.02–0.23	0.03–0.27	−0.16–0.11
*β*	0.074	−0.038	0.112	–	–	–	0.140*	0.163*	−0.027
Parent–adolescent discrepancies in conflict intensity									
M–A discrepancies intercept									
B	0.032	0.071	−0.039	0.018	0.085	−0.067	–	–	–
C.I.	−0.07–0.13	−0.03–0.17	−0.14–0.07	−0.08–0.11	−0.02–0.19	−0.18–0.05	–	–	–
*β*	0.057	0.125	−0.068	0.029	0.141	−0.106	–	–	–
F–A discrepancies intercept									
B	0.083	0.120	−0.037	–	–	–	−0.135	−0.089	−0.046
C.I.	−0.01–0.17	0.03–0.21	−0.13–0.06	–	–	–	−0.22–0.05	−0.19–0.01	−0.15–0.06
*β*	0.130	0.188**	−0.058	–	–	–	−0.205**	−0.132	−0.070

*U* Undercontroller, *R* Resilient, *O* Overcontroller, *AM* adolescent report for mother, *MA* mother report for adolescent, *AF* adolescent report for father, *FA* father report for adolescent, *M-A* discrepancies between the adolescent–mother, and mother–adolescent reports, *F-A* discrepancies between the adolescent–father, and father–adolescent reports *C.I.* confidence intervals

*p < 0.05; **p < 0.01; ***p < 0.001

Regarding adolescent personality, the intercept of adolescent-reported conflict intensity with mothers and with fathers was higher for undercontrolling and for overcontrolling adolescents than for Resilients. Undercontrolling adolescents had a higher intercept of conflict intensity reported by mothers than Resilients. Finally, father–adolescent discrepancies were larger for overcontrolling adolescents than for resilient adolescents.

When considering maternal personality, the intercepts of both mother- and adolescent-reported conflict intensity were significantly higher for undercontrolling and overcontrolling mothers than for resilient mothers. No differences were found between undercontrolling and overcontrolling mothers. Also, no significant differences emerged on mother–adolescent discrepancies.

For paternal personality, the intercept of father-reported conflict intensity was significantly higher for undercontrolling and overcontrolling fathers than for resilient fathers. The intercept of father–adolescent discrepancies was lower for undercontrolling fathers than for resilient fathers, indicating that undercontrolling fathers reported higher conflict compared to their adolescents, thus, larger discrepancies (the overall intercept was zero, see Table 2).

### Sensitivity Analysis

Given gender differences in parent–adolescent relationship quality (De Goede et al. 2009), and the possible influence of family socioeconomic status on parent–adolescent relationship quality, additional analyses were run in which the intercept and the two slopes of each model were regressed on gender and SES. The regression coefficients of the intercepts and slopes on gender and SES were mostly non-significant, except for mother-reported conflict intensity. In that case, gender and SES had a significant effect on the intercept only, indicating that mothers of girls and mothers in lower-SES families reported a higher level of conflict intensity. As can be seen in Table S6 and Table S7 of the supplementary information, 4 out of the 18 means in the latent growth models (research questions 1 and 2), and 2 out of the 36 regression coefficients regarding the effect of personality types (research question 3) changed significance. However, the effect sizes did not change substantially, indicating that including covariates increased the standard errors.

The following differences emerged in the models that controlled for gender and SES, compared to the models without covariates. Father-reported conflict intensity did not show a significant decrease from middle-to-late adolescence. Similarly, mother–adolescent discrepancies did not increase significantly during early-to-middle adolescence, but they did increase significantly from middle-to-late adolescence. Father–adolescent discrepancies did not increase further in middle-to-late adolescence. Finally, the effect of undercontrolling adolescents on mother-reported conflict intensity and the effect of overcontrolling adolescents on adolescent-reported conflict intensity with fathers turned non-significant.

## Discussion

Parent–adolescent relationships tend to be characterized by conflicts. The intensity of those conflicts can be perceived differently by parents and adolescents. Conflict intensity and discrepancies in the perceptions of parents and adolescents might reflect the restructuring of the parent–adolescent relationship that takes place during this period (e.g., De Los Reyes and Ohannessian 2016). In addition, adolescents’ and parents’ personality types might affect how the parent–adolescent relationship transforms. Past research has shown that according to adolescents, conflict intensity changes curvilinearly across adolescence (De Goede et al. 2009), but the views of parents are often overlooked. Given the significance of parent–adolescent conflict intensity and discrepancies in parents’ and adolescents’ perceptions for adolescent adjustment (e.g., Branje 2018), the current study examined the trajectories of parent–adolescent conflict intensity as perceived by adolescents, mothers, and fathers, as well as the trajectories of the discrepancies in perceptions of conflict intensity. Additionally, the current study examined the role of parental and adolescent personality types in these trajectories.

### Development of Parent–Adolescent Conflict Intensity across Adolescence

By addressing mothers’, fathers’, and adolescents’ perceptions of conflict intensity, the results of this study add to the knowledge that has resulted from past research regarding conflict intensity trajectories. First, in agreement with some previous studies (De Goede et al. 2009; McGue et al. 2005), and contrary to others (Galambos and Almeida 1992), this study found that adolescent-reported conflict intensity increases from early to middle adolescence. Second, by following the same adolescents beyond middle adolescence, this study showed that adolescent-reported conflict intensity is stable from middle to late adolescence. Third, and most importantly, this study showed that the trajectories of conflict intensity differed among parents and adolescents, such that parents perceived initially stable, and then declining levels of conflict intensity. These results offer more clarity to the developmental trajectories of parent–adolescent conflict intensity by showing what trend is perceived by whom.

In agreement with the theoretically expected gap in parents’ and adolescents’ expectations for autonomy (Deković et al. 1997), adolescents perceived interactions with their parents as increasingly negative from early to middle adolescence, while parents experienced stable levels of negativity. Given that conflict intensity assessed the mutual negativity in the dyadic relationship, and not specifically how annoyed adolescents get with their parents, or parents with adolescents, this increasing adolescent-reported conflict intensity implies that there are factors that affect adolescents’ perceptions. For example, recent evidence linking increasing parent–adolescent conflicts with pubertal timing and tempo supports this notion (Marceau et al. 2015).

The results of the current study suggest that in the perceptions of adolescents, the intensity of conflict remains stable from middle to late adolescence, and stays higher than in parents’ perceptions, which reflected a decrease in conflict intensity. These findings are in line with earlier findings that adolescents perceived higher conflict engagement than parents (Van Doorn et al. 2011). The results show that the improvement of parent-child relationship quality is not reflected similarly in adolescents’ and parents’ perceptions. The decrease in conflict intensity after middle adolescence as perceived by parents might be a sign of relationship improvement. But at the same time, adolescent-perceived levels remained stable, and they were still higher compared to before middle adolescence. Therefore, relationship improvement is not reflected in adolescent-perceived conflict intensity. Given that adolescents become more autonomous from parents after middle-adolescence (Hadiwijaya et al. 2017), parents might perceive this as relationship improvement, but adolescents still feel that there is an elevated tension in the relationship with their parents.

### Development of Parent–Adolescent Discrepancies in Conflict Intensity across Adolescence

In agreement with theoretical views on the change in parent–adolescent relationship during adolescence (Collins and Laursen 1992), increasing discrepancies from early to middle adolescence were found. This finding concurs with the notion that during adolescence, a needs-opportunities mismatch emerges (Eccles et al. 1993), which leads to increasing parent–adolescent discrepancies. Besides, the fact that parent-perceived conflict intensity is lower than adolescents’ perceptions indicates that adolescents’ increasing frustration is not fully outed. Indeed, poor communication is one reason for discrepant parent–adolescent perceptions (De Los Reyes et al. 2016; Ehrlich et al. 2016). Even though overall low negativity prevails, parents and adolescents hold all the more diverging views on how much negativity exists in their relationship.

The results concur with existing theoretical accounts (e.g., De Los Reyes and Ohannessian 2016), and empirical research (De Haan et al. 2017) that discrepancies are useful to explore further because they are not measurement error. Measurement error was taken into account in the current study by applying a latent-variable technique (Córdova et al. 2016). Additionally, if discrepancies were random error, then the meaningful longitudinal patterns that were found in this study would be unlikely to emerge (De Los Reyes and Kazdin 2005).

The current study offers empirical support to the proposition of the Operations Triad Model (De Los Reyes and Ohannessian 2016), which stipulates that diverging views on family-related concepts reflect evolving family dynamics. In agreement with this proposition, the divergence in parent–adolescent perceptions found in this study indicates two underlying processes in the family. The increasing discrepancies in early-to-middle adolescence were mainly due to the increasing intensity of conflict as perceived by adolescents. The further divergence from middle-to-late adolescence in the father–adolescent relationship could mostly be attributed to the decreasing negativity as perceived by fathers. Thus, these results show that the restructuring of the parent–adolescent relationship (Hadiwijaya et al. 2017) is not just a reflection of adolescent maturation, but also change in parental views.

As noted recently (De Los Reyes et al. 2019), a developmental approach to discrepancies can help elucidate whether increased discrepancies reflect normative parent–adolescent dynamics during adolescence or risk. The average trajectories found in the current study show that increased parent–adolescent discrepancies seem to be normative. However, given that discrepancies have been shown to have negative repercussions for adolescent adaptation (Nelemans et al. 2016), the significant variance around the increasing divergence found in the current study might imply a threat to the relationship, for some dyads. Like a double-edged sword, the decrease in father-perceived conflict intensity and the concomitant increase in discrepancies might prove a threat for some father–adolescent dyads. Future research examining the co-development of parent–adolescent discrepancies with parent and adolescent adaptation may help elucidate this possibility.

### Personality Typologies and Development of Parent–Adolescent (Discrepancies in) Conflict Intensity

Adolescent and parental personality significantly and meaningfully predicted differences in both the trajectories of parent–adolescent conflict intensity and the trajectories of parent–adolescent discrepancies in conflict intensity. As expected, resilient adolescents, mothers, and fathers reported the lowest levels of conflict intensity, whereas resilient adolescents, and fathers also showed the lowest discrepancies. These results support the hypothesis that individual differences, operationalized as personality types, explain differences in how much negativity parents and adolescents experience in their relationship, as well as how differently they perceive their relationship (Belsky 1984).

Across reporters, personality type had an effect on self-reported conflict intensity (“actor effects”). Compared to Overcontrollers and Underconrtollers, resilient adolescents, mothers, and fathers perceived lower conflict intensity, which is in agreement with extant research (Denissen et al. 2007). Having a more flexible personality type allows individuals to more easily adapt to contextual demands (Block and Block 1980), and might as such be associated with less intense conflictual interactions.

Furthermore, personality type also had an effect on the conflict intensity as perceived by the partner (“partner effects”), but this pattern held only for the mother–adolescent dyad. Specifically, mothers perceived undercontrolling adolescents as the most aggravating, implying that having a “difficult” adolescent child is more emotionally demanding for the mothers. This finding is in agreement with studies showing, for example, that parent–adolescent relationships tend to be worse in families in which adolescents have more internalizing or externalizing problems (Crocetti et al. 2016), or difficulties in their identity development (Crocetti et al. 2017). Other studies, however, failed to find an effect of having an “easy” versus a difficult adolescent on parenting (de Haan et al. 2012). Similarly, adolescents with resilient mothers perceived lower conflict intensity than adolescents with overcontrolling or undercontrolling mothers. Thus, having an undercontrolling or an overcontrolling mother poses a challenge in adolescents, as it leads them to experience higher negativity, compared to having a resilient mother. The fact that no partner effects emerged in the father–adolescent dyad indicates that the level of frustration fathers and adolescents perceive in their relationship does not depend on the dyadic partner’s personality.

In addition, personality types were also associated with discrepancies, yet only in father–adolescent dyads. In dyads with overcontrolling adolescents, compared to dyads with a resilient adolescent, the divergence among adolescent and paternal perceptions of conflict intensity was higher. Perhaps overcontrolling adolescents do not express as openly their negative emotions in their relationship with their father, leaving fathers less aware of the negativity in their relationship. Additionally, paternal personality type made a difference in father–adolescent conflict intensity discrepancies. Being an undercontrolling father had a negative impact on father–adolescent discrepancies compared to being a resilient father, indicating that in these dyads fathers reported more conflict intensity than adolescents. Because, on average, the intercept of father–adolescent discrepancies was zero, the negative coefficient found for undercontrolling fathers indicates that this type is associated with higher discrepancies, compared to resilient fathers. Given the detrimental effects specifically of father–adolescent divergence for adolescent adaptation (e.g., Nelemans et al. 2016), this finding means that for adolescents with an undercontrolling father the transition to a more egalitarian parent–adolescent relationship likely starts upon a more turbulent basis. Furthermore, that the development of discrepancies does not depend on personality types indicates that, in agreement with the idea of the diverging operations of the Operations Triad Model (De Los Reyes and Ohannessian 2016), the development of parent–adolescent discrepancies in conflict intensity may reflect normative processes of adolescent development, irrespective of individual differences.

Maternal and paternal personality types were associated with parent–adolescent discrepancies differently. Mother–adolescent discrepancies were predicted by neither maternal nor adolescent personality, whereas father–adolescent discrepancies were predicted by both adolescent (being an Overcontroller compared to being Resilient) and paternal (being an Undercontroller, compared to being Resilient) personality. It seems that mother–adolescent discrepancies may reflect normative developmental trends in the mother–adolescent relationship, whereas the father–adolescent discrepancies are more open to other effects. The present study adds to recent research showing that, compared to the maternal role, the paternal role may be more prone to external influences, such as the influence of the interparental relationship (Mastrotheodoros et al. 2019) or the mental health of their wife (Kouros et al. 2014).

### Limitations, Strengths and Future Directions

Some limitations should be taken into account in this study. First, the study consisted of self-reported data. Even though self-report can be a strong method to assess internal states, conflict intensity could also be assessed with observations of parent–adolescent interactions. Using observations along with discrepancies in self-reports might elucidate further aspects of the transforming parent–adolescent relationship. Second, the sample consisted mainly of middle- and upper-middle class Dutch families and results might differ in lower SES families. For example, lower SES families might experience higher levels of stress and might lack coping mechanisms that would allow them to withhold stress from spilling over to create conflict (Conger et al. 2000). Furthermore, the sample comes from the Netherlands, a relatively affluent western country, with low unemployment rates and a good social security network. These may make for a higher level of family well-being compared to other countries. This characteristic, coupled with the upper-middle class SES of this sample, might imply that the generalizability of this study may be limited.

In spite of these limitations, this study offers new insights into the development of parent–adolescent conflict intensity, by using a relatively large, multi-informant, and longitudinal design following families across adolescence. Specifically, this is the first study to investigate the trajectories of conflict intensity across adolescence, taking into account the views of mothers, fathers, and adolescents. Also, this is among the first studies to investigate the trajectories of parent–adolescent discrepancies, across adolescence, while taking into account the personality types of mothers, fathers, and adolescents. Hence, the current study offers new insights into how the parent–adolescent relationship transforms during adolescence.

Future studies could benefit from examining the trajectories of discrepancies in an expanded developmental time-frame, also including the period before and after adolescence. A more comprehensive view of how discrepancies develop could be reached by incorporating mother and father reports, along with child reports for mothers and fathers separately in longitudinal studies of other periods of development. In addition, investigating the development of family relationships from the perspective of different family members along with indices of adaptation might provide a better understanding of the restructuring of the parent–adolescent relationship. Similarly, investigating possible outcomes of the rate of change in discrepancies, controlling for the level of discrepancies, could prove a useful next step in discrepancy research.

## Conclusion

Parent–adolescent conflict intensity is one aspect of adolescence that has attracted much attention from the popular media and the research community alike (Laursen et al. 1998). Conflict is often a mechanism that forges change in the parent–adolescent relationship (Branje 2018), and, therefore, it is important to understand how it develops during adolescence. However, parents and adolescents experience their conflicts differently (Van Lissa et al. 2015), and taking parental and adolescent perceptions into account is necessary to get a comprehensive picture of conflict intensity. By incorporating more than one informant, however, discrepancies arise among the different reports, and these discrepancies can indicate family processes (De Los Reyes and Ohannessian 2016), like the restructuring of the parent–adolescent relationship. This study investigated the trajectories of parent–adolescent conflict intensity across adolescence, according to mothers, fathers, and adolescents. Also, this study examined the trajectories of parent–adolescent discrepancies and the predictive role of parental and adolescent personality in the development of conflict intensity and discrepancies in conflict intensity. The results showed that parents and adolescents hold different views of conflict intensity, and these differences give rise to discrepancies. Conflict intensity increased only according to adolescents. The two cycles of discrepancies that emerged indicate that the restructuring of the parent–adolescent relationship is not only a matter of adolescent maturation but a matter of parent–adolescent alignment. The level of conflict intensity in the parent–adolescent relationship was lower in families with resilient parents or adolescents, implying that the way toward parent–adolescent alignment might be shorter for families with resilient parents or adolescents. These findings have implications for understanding adolescence, giving insights into the processes of re-alignment of the parent–adolescent relationship. In the process of re-alignment, parent–adolescent discrepancies can be normative, with adolescents feeling more frustrated than parents, also during late adolescence.

## Supplementary information

Supplementary Information
