# Supplementary material for: Parent–Adolescent Conflict across Adolescence: Trajectories of Informant Discrepancies and Associations with Personality Types
Source: J Youth Adolesc. 2019 Jun 26;49(1):119–35. doi: 10.1007/s10964-019-01054-7 (PMC6987059; doi:10.1007/s10964-019-01054-7)
Supplement: Supplementary file 1 — Supplementary Information [file 10964_2019_1054_MOESM1_ESM.docx]

*Table S1*

|  | *χ*^2^ | *df* | CFI | TLI | RMSEA | SRMR | BIC | *Δ*CFI | *Δ*TLI | *Δ*RMSEA |  |
| --- | --- | --- | --- | --- | --- | --- | --- | --- | --- | --- | --- |
|  | Wave 1 | | | | | | | | | | |
| Configural | 161 | 36 | .973 | 0.956 | .085 | .025 | 16951 |  |  |  |  |
| Metric | 212 | 51 | .965 | 0.959 | .082 | .064 | 16917 | .008 | -.003 | .003 |  |
| Scalar | 262 | 66 | .958 | 0.962 | .079 | .060 | 16847 | .007 | -.003 | .003 |  |
|  | Wave 2 | | | | | | | | | | |
| Configural | 177 | 36 | .972 | .953 | .093 | .023 | 16060 |  |  |  |  |
| Metric | 204 | 51 | .970 | .964 | .081 | .044 | 15981 | .002 | -.011 | .012 |  |
| Scalar | 279 | 66 | .958 | .961 | .085 | .041 | 15946 | .012 | .003 | -.004 |  |
|  | Wave 3 | | | | | | | | | | |
| Configural | 131 | 36 | .976 | .960 | .078 | .023 | 15670 |  |  |  |  |
| Metric | 188 | 51 | .966 | .960 | .078 | .060 | 15626 | .010 | .000 | .000 |  |
| Scalar | 250 | 66 | .954 | .958 | .080 | .054 | 15579 | .012 | .002 | -.002 |  |
|  | Wave 4 | | | | | | | | | | |
| Configural | 168 | 36 | .970 | .951 | .093 | .026 | 15165 |  |  |  |  |
| Metric | 200 | 51 | .966 | .960 | .083 | .041 | 15077 | .004 | -.009 | .010 |  |
| Scalar | 268 | 66 | .955 | .959 | .085 | .044 | 15034 | .011 | .001 | .002 |  |
|  | Wave 5 | | | | | | | | | | |
| Configural | 250 | 36 | .951 | .918 | .121 | .030 | 14136 |  |  |  |  |
| Metric | 326 | 51 | .937 | .926 | .115 | .070 | 14117 | .014 | -.008 | .006 |  |
| Scalar | 397 | 66 | .924 | .931 | .111 | .065 | 14063 | .013 | -.005 | .004 |  |
|  | Wave 6 | | | | | | | | | | |
| Configural | 158 | 36 | .973 | .955 | .092 | .024 | 13758 |  |  |  |  |
| Metric | 226 | 51 | .961 | .955 | .092 | .066 | 13725 | .012 | .000 | .000 |  |
| Scalar | 285 | 66 | .952 | .956 | .091 | .062 | 13673 | .009 | -.001 | .001 |  |
|  | Longitudinal Invariance (W1 – W6) | | | | | | | | | |  |
| Configural | 3515 | 1956 | .963 | .953 | .040 | .035 | 70167 |  |  |  |  |
| Metric | 3701 | 2056 | .961 | .953 | .040 | .042 | 69628 | .002 | .000 | .000 |  |
| Scalar | 3992 | 2156 | .957 | .950 | .042 | .044 | 69194 | .004 | .003 | .002 |  |

Fit indices of measurement invariance analyses for the Negative Interactions scale, across adolescent-mother, adolescent-father, mother-adolescent, and father-adolescent reports for waves 1-6, as well as longitudinally across waves 1-6.

*Note:* CFI: Comparative Fit Index; TLI: Tucker-Lewis Index; RMSEA: Root-Mean Square Error of Approximation; SRMR: Standardized Root-Mean Residual; BIC*:* Bayesian Information Criterion. W1-W6: Wave 1 to Wave 6*.*

Table S2

Fit Indices for the Latent Growth Curve Models of Conflict Intensity and Discrepancies in Conflict Intensity with Linear, Quadratic, and Piecewise Specification.

|  | *χ^2^* | | |  | *p* | | |  | CFI | | |  |  | RMSEA | | |  | SRMR | | |
| --- | --- | --- | --- | --- | --- | --- | --- | --- | --- | --- | --- | --- | --- | --- | --- | --- | --- | --- | --- | --- |
|  | Linear | Piecewise* | Quad. |  | Linear | Piecewise | Quad. |  | Linear | Piecewise | Quad. |  |  | Linear | Piecewise | Quad. |  | Linear | Piecewise | Quad. |
| Conflict Intensity |  |  | | | | | |  |  |  |  |  |  |  |  |  |  |  |  |  |
| AM | 84.1 | 38.1 | 35.2 |  | .000 | .001 | .001 |  | .946 | .981 | .983 |  |  | .069 | .042 | .037 |  | .102 | .073 | .067 |
| AF** | 97.7 | 35.7 | 41.7 |  | .000 | .001 | .000 |  | .937 | .983 | .979 |  |  | .072 | .036 | .042 |  | .105 | .095 | .094 |
| MA | 71.8 | 48.2 | 55.8 |  | .000 | .000 | .000 |  | .967 | .979 | .974 |  |  | .059 | .050 | .056 |  | .070 | .073 | .069 |
| FA | 131.2 | 58.2 | 66.7 |  | .000 | .000 | .000 |  | .922 | .970 | .964 |  |  | .101 | .065 | .074 |  | .138 | .109 | .088 |
| Discrepancies |  |  |  |  |  |  |  |  |  |  |  |  |  |  |  |  |  |  |  |  |
| A-M | 87.5 | 27.8 | 46.7 |  | .000 | .010 | .000 |  | .903 | .980 | .955 |  |  | .080 | .037 | .057 |  | .098 | .042 | .056 |
| A-F | 36.7 | 15.5 | 19.5 |  | .004 | .344 | .145 |  | .977 | .998 | .994 |  |  | .035 | .000 | .009 |  | .057 | .043 | .046 |

Note: AM: Adolescent report for mother; AF: adolescent report for father; MA: Mother report for adolescent; FA: Father report for adolescent. CFI: Comparative Fit Index; RMSEA: Root-Mean Square Error of Approximation; SRMR: Standardized Root-Mean Residual. *For all the piecewise models, the knot was set to Wave 4 (adolescent age 16 years), except if otherwise specified. **For this piecewise model, the knot was set to Wave 3 (adolescent age 15 years).

*Table S3*

Parameter Estimates (means and variances) of the Latent Congruency Models for the Discrepancies, Across Waves.

|  | Mother-Adolescent | | Father-Adolescent | |
| --- | --- | --- | --- | --- |
|  | Mean | Variance | Mean | Variance |
| Wave 1 | 0.148*** | .313*** | 0.004 | .229*** |
| Wave 2 | 0.159*** | .296*** | 0.075** | .281*** |
| Wave 3 | 0.211*** | .279*** | 0.151*** | .257*** |
| Wave 4 | 0.231*** | .344*** | 0.152*** | .291*** |
| Wave 5 | 0.305*** | .379*** | 0.184*** | .285*** |
| Wave 6 | 0.255*** | .275*** | 0.218*** | .374*** |

*Note*: **p* < .05; ***p* < .01; ****p* < .001.

Table S4

Means and 95% Confidence Intervals for Growth Parameters of the Adolescent Big Five, for Resilients, Overcontrollers, and Undercontrollers.

| Adolescent Personality Types | Resilients (*n* = 183, 36.8%) | Overcontrollers (*n* = 156, 31.4%) | Undercontrollers (*n* = 158, 31.8%) |
| --- | --- | --- | --- |
|  | *M* (95% C.I.) | *M* (95% C.I.) | *M* (95% C.I.) |
| Intercepts |  |  |  |
| Ag | 5.83***^a^ (5.74, 5.91) | 5.51***^b^ (5.41, 5.60) | 5.02***^c^ (4.89, 5.15) |
| ES | 4.71***^a^ (4.57, 4.85) | 3.73***^b^ (3.56, 3.89) | 4.81***^a^ (4.68, 4.94) |
| Ext | 5.57***^a^ (5.43, 5.70) | 4.34***^c^ (4.20, 4.48) | 5.23***^b^ (5.11, 5.36) |
| Con | 4.19***^a^ (4.02, 4.36) | 4.20***^a^ (4.09, 4.42) | 3.54*** ^b^ (3.39, 3.68) |
| Op | 5.40***^a^ (5.29, 5.51) | 5.07***^b^ (4.95, 5.19) | 4.08***^c^ (3.96, 4.21) |
| Linear Slopes |  |  |  |
| Ag | -.069 (-.186, .047) | -.115 (-.264, .033) | -.264** (-.446, -.082) |
| ES | .157** (.054, .260) | -.110 (-.236, .015) | .171** (.055, .287) |
| Ext | .138** (.038, .238) | -.150** (-.256, -.044) | .143** (.042, .245) |
| Con | -.013 (-.114, .088) | .062 (-.037, .161) | -.102 (-.209, .004) |
| Op | .065 (-.009, .139) | -.016 (-.103, .071) | .022 (-.087, .131) |
| Quadratic Slopes |  |  |  |
| Ag | .054 (-.002, .109) | .071 (-.002, .144) | .164*** (.078, .250) |
| ES | -.026** (-.045, -.007) | .020 (-.003, .043) | -.035* (-.056, -.013) |
| Ext | -.020* (-.038, -.002) | .018 (-.001, .038) | -.033** (-.052, -.013) |
| Con | .004 (-.015, .022) | -.011 (-.029, .008) | .029** (.009, .048) |
| Op | -.012 (-.025, .002) | -.003 (-.018, .012) | .005 (-.015, .025) |
| Cubic Slopes |  |  |  |
| Ag | -.007* (-.014, -.000) | -.009 (-.019, .001) | -.021*** (-.032, -.010) |

Note: Ag: Agreeableness; ES: Emotional Stability; Ext: Extraversion; Con: Conscientiousness; Op: Openness; C.I.: Confidence Intervals. Values with different letter indicators indicate statistically different means.

**p* < .05; ***p* < .01; ****p* < .001.

Table S5

Means and 95% Confidence Intervals for Growth Parameters of the Parental Big Five, for Resilients, Overcontrollers, and Undercontrollers.

| Parental Personality Types | Resilients (*n* = 456, 47.8%) | Overcontrollers (*n* = 253, 26.5%) | Undercontrollers (*n* = 245, 25.7%) |
| --- | --- | --- | --- |
|  | *M* (95% C.I.) | *M* (95% C.I.) | *M* (95% C.I.) |
| Intercepts |  |  |  |
| Ag | 5.92***^a^ (5.88, 5.97) | 5.40***^c^ (5.33, 5.47) | 5.56***^b^ (5.48, 5.64) |
| ES | 5.08***^a^ (4.99, 5.16) | 4.16***^c^ (4.03, 4.28) | 4.60***^b^ (4.48, 4.72) |
| Ext | 5.50***^a^ (5.42, 5.78) | 3.73***^c^ (3.63, 3.82) | 4.93***^b^ (4.81, 5.05) |
| Con | 5.52***^a^ (5.45, 5.58) | 5.28***^b^ (5.18, 5.38) | 3.76*** ^c^ (3.67, 3.86) |
| Op | 5.04***^a^ (4.96, 5.13) | 4.12***^c^ (4.00, 4.24) | 4.83***^b^ (4.71, 4.96) |
| Linear Slopes |  |  |  |
| Ag | .022*** (.013, .031) | .008 (-.006, .022) | .008 (-.007, .022) |
| ES | .069***^a^ (.054, .083) | .027*^b^ (.005, .049) | .044***^b^ (.022, .066) |
| Ext | .038*** (.023, .053) | .014 (-.005, .032) | .018 (-.001, .037) |
| Con | .039*** (.026, .052) | .005 (-.012, .023) | .025** (.006, .044) |
| Op | .009 (-.004, .023) | -.007 (-.024, .010) | .001 (-.015, .016) |

Note: Ag: Agreeableness; ES: Emotional Stability; Ext: Extraversion; Con: Conscientiousness; Op: Openness; C.I.: Confidence Intervals. Values with different letter indicators imply statistically different means.

**p* < .05; ***p* < .01; ****p* < .001.


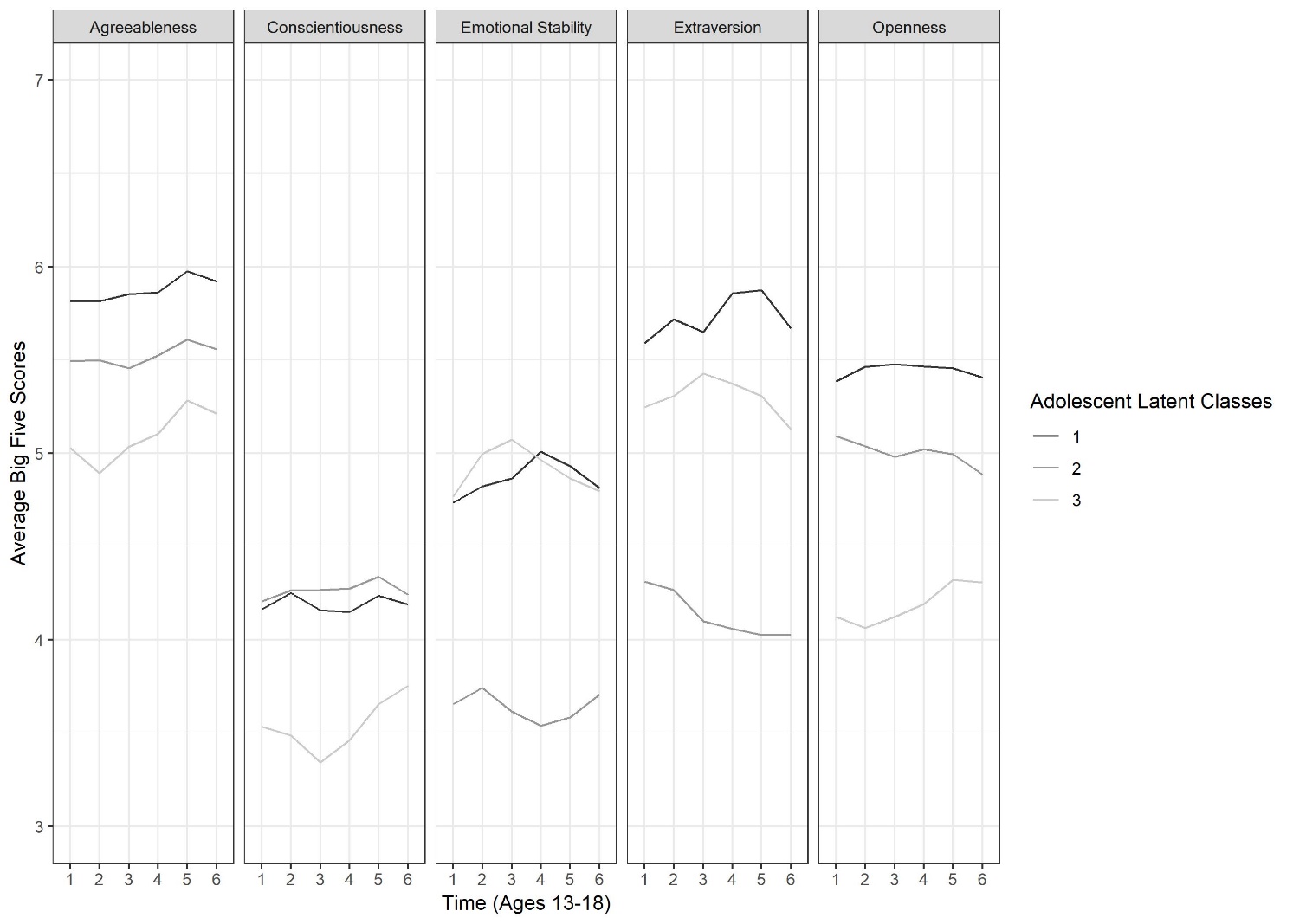
Figure S1. Means on the Big Five in the 3-class solution from the adolescent Latent Class Growth Analysis.


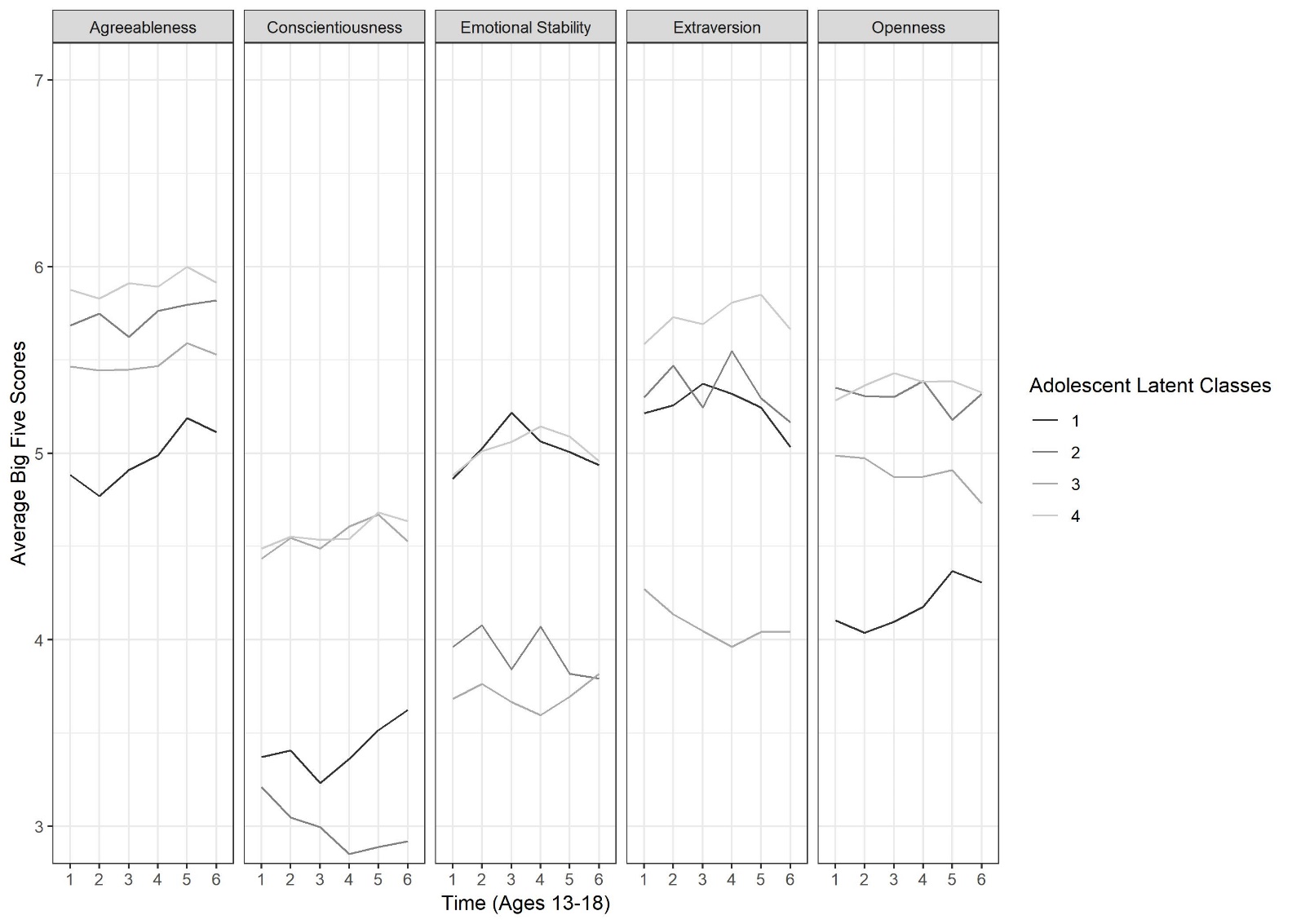


Figure S2. Means on the Big Five in the 4-class solution from the adolescent Latent Class Growth Analysis.

**
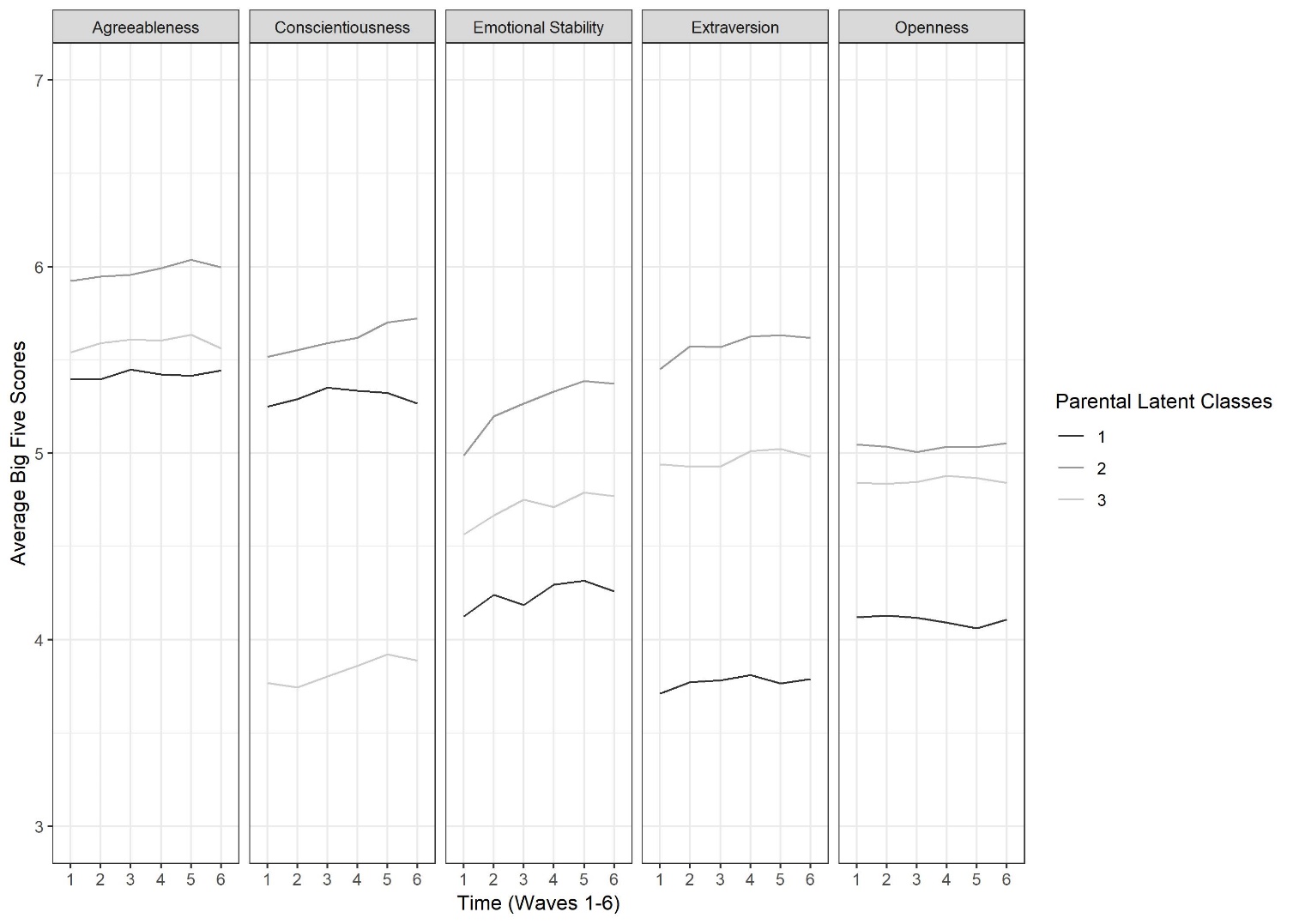
**

Figure S3. Means on the Big Five in the 3-class solution from the parental Latent Class Growth Analysis.

**
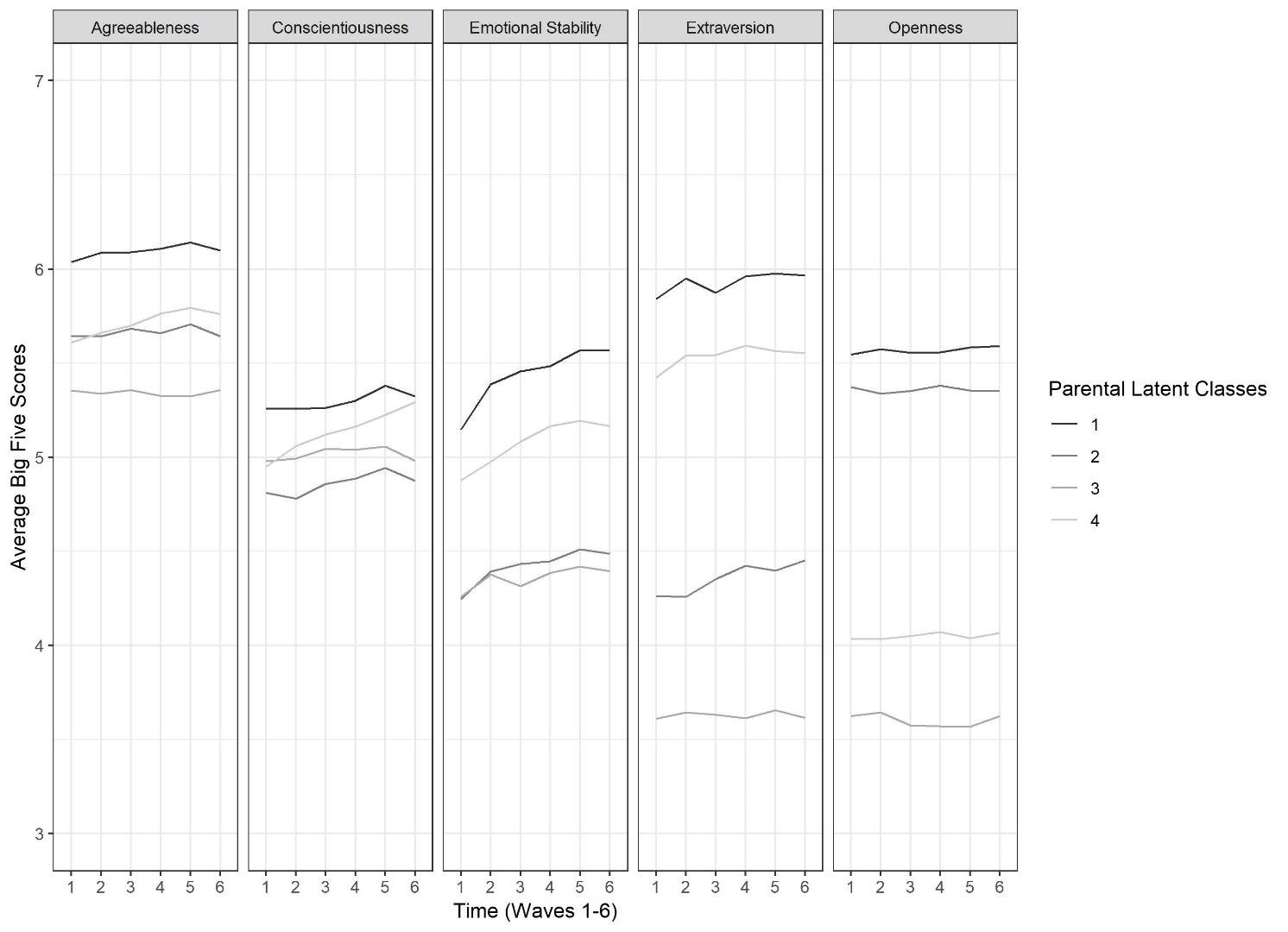
**

Figure S4. Means on the Big Five in the 4-class solution from the parental Latent Class Growth Analysis.

*Table S6*

Growth Parameter Estimates (Means and Variances) of the Latent Growth Curve Models for Adolescent-, Mother-, and Father-reported Conflict Intensity, and the Latent Growth Curve Models for Mother-Adolescent and Father-Adolescent Discrepancies in Conflict Controlling for Gender and Family SES.

|  | Intercept | | Slope 1 | | Slope 2 | |
| --- | --- | --- | --- | --- | --- | --- |
|  | Mean | Variance | Mean | Variance | Mean | Variance |
| Conflict Intensity |  |  |  |  |  |  |
| AM | 1.60*** | .190*** | .060** | .031** | -.001 | .015** |
| AF | 1.51*** | .210*** | .069*** | .034*** | .005 | .026*** |
| MA | 1.44*** | .154*** | .027 | .010** | -.020* | .006** |
| FA | 1.50*** | .154*** | .015 | .013** | -.017 | .016*** |
| Discrepancies |  |  |  |  |  |  |
| Mother-Adolescent | 0.167*** | 0.072*** | 0.024 | 0.018*** | 0.022* | 0.009** |
| Father-Adolescent | 0.004 | 0.084*** | 0.060*** | 0.011** | 0.018 | 0.010*** |

*Note*: AM: Adolescent report for mother; AF: adolescent report for father; MA: Mother report for adolescent; FA: Father report for adolescent.

**p* < .05; ***p* < .01; ****p* < .001.

*Table S7*

Regression Coefficients (Unstandardized *Β*, and Standardized *β*) and Confidence Intervals for the Prediction of the Intercepts of Conflict Intensity and Discrepancies in Conflict Intensity by Adolescent and Parental Personality Type, Controlling for SES and Gender.

|  |  | Adolescent | | |  | Mother | | |  | Father | | |
| --- | --- | --- | --- | --- | --- | --- | --- | --- | --- | --- | --- | --- |
|  |  | U vs. R | O vs. R | U vs. O |  | U vs. R | O vs. R | U vs. O |  | U vs. R | O vs. R | U vs. O |
|  |  | Parent-Adolescent Conflict Intensity | | | | | | | | | | |
| AM Intercept | B | .126 | .126 | .000 |  | .140 | .222 | -.081 |  | - | - | - |
|  | C.I. | .01-.24 | .01-.25 | -.14-.14 |  | .01-.27 | .10-.35 | -.24-.07 |  | - | - | - |
|  | *β* | .132* | .132* | -.001 |  | .132* | .220*** | -.077 |  | - | - | - |
|  |  |  |  |  |  |  |  |  |  |  |  |  |
| MA Intercept | B | .094 | .049 | .045 |  | .152 | .125 | .026 |  | - | - | - |
|  | C.I. | -.01-.20 | -.06-.15 | -.07-.16 |  | .04-.26 | .02-.23 | -.10-.16 |  | - | - | - |
|  | *β* | .108 | .057 | .051 |  | .158** | .138* | .028 |  | - | - | - |
|  |  |  |  |  |  |  |  |  |  |  |  |  |
| AF Intercept | B | .140 | .101 | .039 |  | - | - | - |  | -.009 | .048 | -.057 |
|  | C.I. | .02-.26 | -.02-.23 | -.09-.17 |  | - | - | - |  | -.13-.11 | -.08-.18 | -.20-.09 |
|  | *β* | .141* | .102 | .039 |  | - | - | - |  | -.009 | .046 | -.056 |
|  |  |  |  |  |  |  |  |  |  |  |  |  |
| FA Intercept | B | .065 | -.029 | .094 |  | - | - | - |  | .124 | .150 | -.025 |
|  | C.I. | -.06-.18 | -.13-.07 | -.02-.21 |  | - | - | - |  | .02-.23 | .03-.27 | -.16-.11 |
|  | *β* | .076 | -.035 | .111 |  | - | - | - |  | .141* | .166* | -.029 |
|  | Parent-Adolescent Discrepancies in Conflict Intensity | | | | | | | | | | | |
| M-A Discrepancies Intercept | B | .035 | .070 | -.035 |  | .004 | .080 | -.076 |  | - | - | - |
|  | C.I. | -.06-.13 | -.03-.17 | -.14-.07 |  | -.09-.10 | -.02-.18 | -.19-.04 |  | - | - | - |
|  | *β* | .062 | .124 | -.063 |  | .007 | .134 | -.120 |  | - | - | - |
|  |  |  |  |  |  |  |  |  |  |  |  |  |
| F-A Discrepancies Intercept | B | .068 | .093 | -.026 |  | - | - | - |  | -.119 | -.080 | -.038 |
|  | C.I. | -.02-.16 | .00-.18 | -.12-.07 |  | - | - | - |  | -.21- -032. | -.18-.014 | -.14-.062 |
|  | *β* | .108 | .149* | -.041 |  | - | - | - |  | -.182** | -.121 | -.059 |

*Note:* U: Undercontroller; R: Resilient; O: Overcontroller; AM: Adolescent report for Mother; MA: Mother report for Adolescent; AF: Adolescent report for Father; FA: Father report for Adolescent; M-A: Discrepancies between the Adolescent for Mother, and Mother for Adolescent reports; F-A: Discrepancies between the Adolescent for Father, and Father for Adolescent reports. C.I.: Confidence Intervals.

**p* < .05; ***p* < .01; ****p* < .001.
